# General anesthesia for treating scoliosis with congenital myasthenia syndrome: a case report

**DOI:** 10.1186/s40981-022-00560-1

**Published:** 2022-08-31

**Authors:** Atsushi Yamashita, Yuka Muramatsu, Hiromi Matsuda, Hirotsugu Okamoto

**Affiliations:** Department of Anesthesia, Kitasato University School of Mediceine, 1-15-1 Kitasato, Sagamihara, Kanagawa 252-0375 Japan

**Keywords:** Congenital myasthenia syndrome, Neuromuscular transmission monitoring, Non-invasive positive pressure ventilation

## Abstract

**Background:**

Congenital myasthenia syndrome is a heterogeneous disease with impaired neuromuscular transmission.

**Case presentation:**

This report describes a 13-year-old child with congenital myasthenia syndrome who underwent surgery for scoliosis under general anesthesia. We used a small dose of rocuronium, neuromuscular transmission monitoring, and non-invasive positive pressure ventilation for postoperative respiratory management. There were no respiratory complications during the perioperative period.

**Conclusion:**

As there are only a few reports on the anesthetic management of patients with congenital myasthenia syndrome, we applied the principles of managing autoimmune myasthenia gravis. The postoperative management described herein can prevent respiratory complications in patients with congenital myasthenia syndrome.

## Background

Congenital myasthenia syndrome (CMS) is a rare heterogeneous disease with impaired neuromuscular transmission (NMT), giving rise to motor disorders and weakness. Its prevalence is reportedly 9.2 per 1,000,000 children less than 18 years of age [1]. There are only a few reports on general anesthesia for children with CMS, and we found no report on general anesthesia without complications. This case report describes surgery for scoliosis under general anesthesia in a child with CMS.

## Case presentation

Written informed consent was obtained from the patient and his parent.

A 13-year-old child weighing 67 kg presented with scoliosis, and posterior fixation was planned. The child had motor retardation and scoliosis in infancy and was diagnosed with CMS with a mutation in the COLQ gene at 11 years of age. Non-invasive positive pressure ventilation (NPPV) at night had been started at the age of 6 because of recurrent pneumonia and shortness of breath.

Preoperative examination revealed moderate restrictive ventilatory impairment, but no cardiac dysfunction. No premedication was prescribed. An NMT monitor (GE Healthcare, Finland Oy) was placed on the right forearm and calibrated after administering fentanyl and propofol. The control of the train-of-four ratio was 77%. Three minutes after administering 10 mg rocuronium, the train-of-four count decreased to zero. Anesthesia was maintained with a continuous infusion of propofol and remifentanil. Rocuronium was not added and the train-of-four ratio gradually increased to the control level by the end of the procedure. An epidural catheter was placed by the surgeon and continuous levobupivacaine infusion was used for postoperative analgesia. Quick emergence was observed after discontinuing propofol and administering 2 mg/kg of sugammadex. The tidal volume was increased by over 5 mL/kg and the endotracheal tube was removed. The airway reflexes were weak but preserved. NPPV (V60, Phillips, USA) was started immediately after extubation and the patient was transported to the pediatric intensive care unit. The arterial partial pressure of carbon dioxide was 49 mmHg and arterial saturation of oxygen was 99% (fraction of inspired oxygen was 0.21) on arrival at the pediatric intensive care unit. Postoperative pain seemed to be controlled by epidural analgesia, continuous fentanyl infusion, and acetaminophen. NPPV continued all day until postoperative day 2, and there were no perioperative respiratory complications. The child was discharged on postoperative day 17 after rehabilitation without major complications.

## Discussion

CMS is a heterogeneous disease that impairs NMT [1, 2]. The clinical features of CMS are motor disorders, weakness, and muscle atrophy. There are several differences between CMS and autoimmune myasthenia gravis (MG). First, autoimmune MG is an acquired disease caused by autoantibodies against the acetylcholine receptors. In contrast, CMSs are congenital diseases and are caused by various deficiencies of presynaptic-, synaptic interval-, and postsynaptic elements. Second, because of their differences, CMSs require different therapeutic strategies than those used for autoimmune MG.

Many genetic mutations are associated with CMS, which influence its clinical features, severity, and response to treatment. Of the 25 mutations known to cause CMS, most show an autosomal recessive form of inheritance. The most common cause of CMS is a defect in the synaptic interval, especially in the acetylcholine receptor. Mutation in the COLQ gene causes a defect in collagen Q that anchors acetylcholinesterase to the basal lamina, resulting in an extended residence of acetylcholine in the synaptic interval, leading to excessive acetylcholine receptor activation. Ephedrine is effective for treating the clinical symptoms of CMS with COLQ gene mutation, but acetylcholinesterase inhibitors aggravate the phenotype of CMS.

Few reports have described the use of general anesthesia to manage CMS. Emura et al. [3] did not use neuromuscular blockers and continued mechanical ventilation until the day after the operation for scoliosis in patients with CMS. They reported abdominal bloating after NPPV administration in that patient and considered that it was caused by air injection from NPPV because of decreasing lung-thoracic compliance. A few researchers have reported that regional anesthesia can safely be performed in patients with CMS [4]. According to a review by Gilmore, these patients can be managed using experience with treating autoimmune myasthenia gravis [2].

For the anesthetic management of patients with CMS, knowing the CMS phenotype and treatment is necessary. For example, acetylcholinesterase inhibitors can worsen some CMS phenotypes, while improving others. Volatile anesthetic agents reportedly decrease the neuromuscular function in autoimmune myasthenia gravis. McBeth et al. reported the use of isoflurane for sedating a patient with CMS, concluding that using 1–2.5% isoflurane is a safe and easy method of sedation that avoids the use of a neuromuscular blocking agent [5]. The safety of propofol and midazolam is unclear, but we presumed that they can safely be used in patients with CMS, similar to their use in treating autoimmune myasthenia gravis. Succinylcholine is expected to worsen the existing state of excitotoxicity in some CMS phenotypes and should be avoided in patients with CMS. CMS patients have an exaggerated response to nondepolarizing neuromuscular blockers, but an acetylcholinesterase inhibitor used as a medication for some phenotypes of CMS may decrease the response to such blockers. Therefore, nondepolarizing neuromuscular blockers should be titrated with NMT monitoring.

Postoperative respiratory management of patients with CMS is difficult. NPPV may be beneficial against respiratory dysfunction and the effect of residual anesthetic agents. We extubated in the operating room in this case because the pediatric intensive care unit of our institution is used for respiratory management and NPPV. Nevertheless, invasive positive pressure ventilation in the early postoperative period should be considered for each patient and institution.

Postoperative analgesia is very important for patients after scoliosis surgery. However, patients with CMS have compromised respiratory functions, and using opioids in them may pose a greater risk of adverse events. Choi et al. reported that administering opioids (fentanyl or oxycodone) reduces airway reflex at tracheal extubation in patients undergoing cholecystectomy but reported no postoperative respiratory complications [6]. Moreover, a continuous infusion of fentanyl may increase the risks of weakness of airway reflex and respiratory depression. Therefore, reducing opioids by introducing multimodal analgesia (using opioids with non-steroidal anti-inflammatory drugs, acetaminophen, and regional analgesia) should be considered in patients with CMS, which has also been an important consideration in patients with MG [7]. Additionally, rigorous monitoring in the ICU is needed until the patients fully recover to reach their preoperative respiratory conditions.

## Data Availability

Not applicable.

## Abbreviations

CMS: Congenital myasthenia syndrome
NMT: Neuromuscular transmission
NPPV: Non-invasive positive pressure ventilation
